# EEN Yesterday and Today … CDED Today and Tomorrow

**DOI:** 10.3390/nu12123793

**Published:** 2020-12-10

**Authors:** Marta Herrador-López, Rafael Martín-Masot, Víctor Manuel Navas-López

**Affiliations:** Pediatric Gastroenterology and Nutrition Unit, Hospital Regional Universitario de Málaga, 29011 Málaga, Spain; martahe_93@hotmail.com (M.H.-L.); rafammgr@gmail.com (R.M.-M.)

**Keywords:** Crohn’s disease, CDED, dietary treatment, exclusive enteral nutrition, treatment, children, adults

## Abstract

The treatment of Pediatric Crohn’s Disease (CD) requires attention both to achieve mucosal healing and to optimize growth, while also maintaining proper bone health. Exclusive Enteral Nutrition (EEN) is recommended as first-line treatment in luminal CD. The therapeutic mechanisms of EEN are being discovered by advances in the study of the gut microbiota. Although the total exclusion of a normal diet during the time of EEN continues to be of high importance, new modalities of dietary treatment suggest a successful future for the nutritional management of CD. In this sense, Crohn’s Disease Exclusion Diet (CDED) is a long-term strategy, it apparently acts on the mechanisms that influence the appearance of inflammation (reducing dietary exposure to products negatively affecting the microbiota), but does so using specific available whole foods to achieve this goal, increases the time of clinical remission and promotes healthy lifestyle habits. The development of CDED, which partly minimizes the problems of EEN, has enabled a turnaround in the treatment of pediatric CD. This review highlights the role of enteral nutrition in the treatment of Crohn’s disease with special emphasis on newer dietary modalities such as CDED.

## 1. Introduction

Crohn’s disease (CD) is a chronic idiopathic inflammatory disorder characterized by periods of inflammatory activity that alternate with others of remission. Approximately 10–25% of cases of inflammatory bowel disease (IBD) are diagnosed before 21 years of age [1], with a peak of maximum incidence during childhood between 13–15 years [2,3]. This period is critical [4,5] since important physical changes take place [6] with a rapid growth rate. Growth failure, bone metabolism disorders, delayed puberty, malnutrition, and micronutrient and vitamin deficiencies are frequently associated with pediatric IBD [7]. Even in remission, in IBD children total energy expenditure, active-induced energy expenditure and physical activity are reduced compared to healthy age-matched controls [8]. The growth retardation that occurs early in 40–50% of children with CD may persist into adulthood in up to 15–30% of patients, and approximately 20% of children do not reach their target height. It is interesting to highlight the fact that growth retardation is related to the time from the onset of symptoms to diagnosis, being 3 times higher in those cases that take more than 6 months to be diagnosed [9]. The therapeutic objectives in CD are the control of inflammation, mucosal healing, modification of the course of the disease, avoiding the undesirable effects of treatment and guaranteeing adequate growth and development [10]. The therapeutic arsenal available for the treatment of pediatric CD is very similar to that for adults. Recent evidence suggests that environmental factors and diet in particular may play an important role in the pathogenesis of IBD, with the strongest evidence to date for CD leading to dietary interventions in this type of IBD. Despite the advancement in the therapeutic field brought by biological drugs, exclusive enteral nutrition (EEN) continues to be considered the first-line treatment in luminal pediatric CD [11].

The following are the most relevant aspects that justify the use of EEN as a therapeutic modality in pediatric CD, its possible mechanisms of action and the latest modifications in the classic EEN regimens that augur a promising future for the dietary treatment of pediatric CD, with special emphasis on the Crohn’s Disease Exclusion Diet (CDED).

## 2. The Beginnings of EEN

The use of enteral nutrition in CD runs parallel to the development of enteral formulas. In 1969, the first case of a CD patient treated with enteral nutrition was published, although not with the aim of achieving remission [12]. In 1971 Voitk et al. [13] experimented with treating adults with IBD awaiting surgical intervention with elemental formula, observing clinical improvement and nutritional status and in some cases the remission of symptoms avoiding the surgical intervention they were waiting for. The publication of case series and later the controlled study by O’Morain [14] promoted the resurgence of the use of this therapeutic modality in CD. The first published series in children date also from the early 1980s [15].

## 3. Mechanisms of Diet on Inflammation

IBD is a chronic disorder whose etiopathogenesis is still not well understood, although it is postulated that a defect in the microbiome-host interaction conditioned by environmental factors in genetically predisposed people, leads to an alteration in the innate and acquired immune response. One environmental risk factor that is being investigated in detail is diet. Being able to understand the mechanisms by which diet produces intestinal inflammation allows us to modify this aspect in a simple and effective way. Thus, the different components of the diet can have an important effect on the composition of the microbiota and on the functionality and development of the intestinal immune system. Two facts are clear in relation to the effect of diet on CD: on the one hand, this disease is clearly on the increase in countries exposed to industrialization and the «western diet». On the other hand, EEN (through the exclusion of all types of food) induces clinical remission in the first flare or during relapses [16,17], induces mucosal [18] and transmural [19] healing, has a positive effect on growth [20], on bone health [21], on nutritional status (20), in health-related quality of life [22], and it is a therapeutic option to reduce the risk of relapses during follow-up [23], to avoid treatment with steroids (18) and to update the vaccination schedule for these patients before starting immunosuppressive treatment [24].

Different human and murine models have revealed the deleterious effect of certain dietary components on the structures and mechanisms responsible for intestinal homeostasis (Table 1) and on the microbiome (Table 2).

Several possible mechanisms can be associated with epithelial barrier defects in patients with CD. In the first scenario, intestinal permeability is increased leading to increased exposure to bacterial antigens followed by loss of tolerance and inflammation. A second possible scenario begins with a dietary induced breakdown of the barrier or increase in intestinal permeability favoring bacterial translocation, triggering an adaptive immune response that aims to contain the process. In both scenarios, the components of the diet that are the most likely candidates are those contained in a westernized diet.

Bacterial adherence to the intestinal epithelium, its entry and replication within epithelial cells, dendritic cells, and macrophages leads to continuous stimulation of the adaptive immune system and inflammation [25,26]. If the patient also has a genetic predisposition, carrying genes responsible for the loss of autophagy or Paneth cell dysfunction, the innate immune system will be unable to detect and eradicate these bacteria, giving rise to a continuous stimulation of the adaptive immune system response, lead to tissue damage, loss of epithelial integrity, and increased entry of bacteria, closing a vicious cycle of inflammation called the “bacterial penetration cycle” [25,26,27,28].

## 4. Mechanism and Efficacy of Exclusive Enteral Nutrition

The therapeutic effects of EEN are likely to be associated with exclusion of harmful factors from the diet as different medical formulas with varying caloric and protein or carbohydrate content can be used for EEN, while exposure to habitual diet after EEN causes a rebound in inflammation. This suggests that dietary components that are not present in EEN may be driving inflammation, either [25,26,49] via a direct anti-inflammatory effect [50] or by inducing changes to the microbiota [51]. In recent years, the study of the microbiota has advanced considerably, mainly due to advances in sequencing technology and complex bioinformatics [51]. The diversity of the microbiota is usually diminished in patients with CD before starting treatment with EEN [52,53,54]. Furthermore, the proven fact that EEN produces a decrease in diversity and reduces supposedly protective bacterial species is a true paradox [53,55,56]. Modifications in the microbiota before and after the first week of EEN are being investigated as possible markers of response in the short and medium term. Another paradox is that the recurrence of CD after EEN is associated with an increase in the diversity of the microbiota [56]. However, it could be that the decrease in diversity, per se, is not driving inflammation, and would only indicate that the reduction of harmful taxa could be more likely a mechanism of response than the alteration of the protective bacteria.

Other metagenomic studies have revealed different alterations in metabolic pathways [49,53]. Dunn et al. [57] describe differences in the microbiota between patients who have a sustained response to EEN and those who relapse early when they start on a free diet. This second group has a high abundance of proteobacteria before the onset of EEN and their proportion does not vary after the period of EEN.

Analysis of published data demonstrate the efficacy of EEN for the treatment of CD (Table 3), a remission rate of 75.7% (CI, 95% 73.8–77.5) is found, although it varies according to remission criteria, duration of EEN or the type enteral formulas used. More than 60 different enteral formula have been used [58]. EEN induces clinical remission in the first flare [59,60] or duringaffect intestinal barrier mechanisms and host immunity relapses [16,17], induces mucosal [18] and transmural [19] healing, has a positive effect on growth [20] and bone health [21], improves nutritional status [61], has a positive effect on health-related quality of life [22] and is a therapeutic option to reduce the risk of relapse during follow-up [23], to avoid treatment with steroids [20,62] and to update the vaccination schedule of these patients before starting immunosuppressive treatment [24].

The efficacy of EEN for the control of successive flares is estimated to vary between 50% and 75% [16,63]. However, although this response rate is lower than when it is use at the onset of disease, a decrease in inflammatory activity and an improvement in nutritional status have been described even in patients who do not reach remission. No differences have been described in the remission or compliance rate in relation to the type of enteral formula used (polymeric, semi-elemental, or elemental), supplemented or not with glutamine or medium chain triglycerides [64]. The formula can be administered by mouth or by nasogastric tube (NGT), since no strategy has been shown to be more effective, although the oral route has many advantages (costs, side effects, and ease of administration), although palatability can influence treatment compliance [65].

Remission rates in the adult population are shown in Table 4. The efficacy of EEN in complicated CD in adults has been studied with good results. A prospective observational study included 41 patients with fistulizing disease, inflammatory strictures, and abscesses. All received EEN, and those with abscesses also received antibiotics with or without surgical drainage. After 12 weeks of treatment, 80.5% of patients achieved clinical remission and 75% of patients with enterocutaneous fistulas experienced fistula closure [66]. In another study, a marked improvement in inflammatory strictures was observed with a 331% reduction in the luminal cross-sectional area assessed by imaging tests [67].

EEN has also gained special relevance in the preoperative treatment of children and adults with CD who are going to undergo surgical resection, improving evolution after surgery [127,128].

## 5. Predictive Factors of Response to Exclusive Enteral Nutrition

Regarding the predictive factors of response, those patients with a mild-moderate flare (wPCDAI < 57.5), with faecal calprotectin < 500 mcg/g of stool, ileal involvement, and C-reactive protein > 15 mg/L showed a better response to the EEN [107]. However, there is still controversy about the efficacy of EEN according to the location of the CD, classically being attributed a worse response in cases of exclusively colonic involvement. Afzal et al. [79] demonstrated that colonic CD did not respond the same as when there is ileal involvement (11/12 patients with L1, 32/39 with L3 and 7/14 with L2, *p* = 0.0021). DeBie et al. [89] found a higher remission rate in patients with exclusive ileal involvement (14/16 with L1 versus 8/15 with L2 and 18/35 with L3, *p* = 0.04). Other investigators [85] have not confirmed the same results (10/13 with L1 **vs.** 15/19 with L2 achieved remission after 4 weeks of EEN, *p* = 0.88). The current recommendation is that EEN should be used in luminal CD regardless of the location, although when there is severe perianal disease, or the presence of deep colonic ulcers, treatment with anti-TNF can be considered as the first therapeutic option [129].

## 6. Complications of Exclusive Enteral Nutrition

EEN is a safe and well-tolerated treatment. An extremely rare side effect is refeeding syndrome [7]. For that reason, in those patients with severe malnutrition who are going to receive EEN is important to carry out a clinical-analytical control in a hospital setting before starting the treatment.

## 7. Quality of Life and Exclusive Enteral Nutrition

The burden of disease imposed on children and adolescents with CD colitis is considerable. However, the psychological burden to which these patients are subjected has been less frequently documented. The instruments for evaluating health-related quality of life (HRQOL) aim to weigh this aspect. The HRQOL can be defined as the degree of general satisfaction with life that is influenced, positively or negatively, by the perception that people have about certain aspects of life that are important to them, including issues related or not to health. Afzal et al. [22] verified an improvement in the HRQOL measured by the IMPACT II (HRQOL questionnaire) of 26 patients with CD after 8 weeks of EEN; this improvement did not correlate with mucosal healing, but it did correlate with a significant decrease in PCDAI. This is because in children with IBD, HRQOL is directly related to inflammatory activity, symptoms and psychological comorbidity. Furthermore, low HRQOL scores are predictive of overuse of health services by patients with IBD.

## 8. Barriers and Facilitating Elements to Use Exclusive Enteral Nutrition. Predictors of Non-Adherence to Exclusive Enteral Nutrition

**Figure 1:**
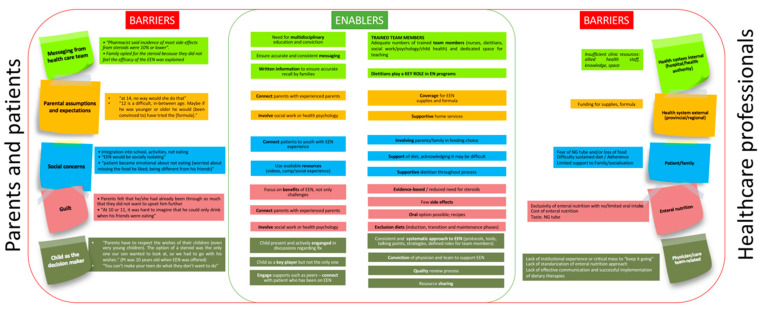
Barriers and facilitators of EEN. Adapted from [130,131,132,133,134].

In a survey carried out in 51 gastroenterology units of the Spanish territory [63] the lack of acceptance by the patient and/or family (71%) and the lack of time and/or allied health staff (dietitians, nutritionists, psychologists, etc.) to collaborate in the follow-up and support of these patients (69%) were the most frequent limiting factors when establishing EEN as a treatment. Other less important aspects were the difficulty in convincing the patient and/or family about the suitability of the treatment (43%), the budget limitation (10%), the difficulty in using alternatives to oral administration like NGT or gastrostomy at hospital (8%), or the logistical difficulties for the prescription and administration of nutrition by the hospital (2%). Although the difficulties are similar in other areas, such as North America [130,131], it is important to highlight that the gastroenterologist’s clinical training as well as health care systems influences the use of EEN in their daily clinical practice. There is a dramatic difference in medical practice, as EEN is not very often used in US while it is the standard in EU for Pediatric CD. Figure 1 shows the barriers and facilitating elements to establish and maintain EEN in patients with CD.

DeBie et al. [89] studied adherence to EEN, non-compliant patients were significantly older (15.5 years vs. 13.4 years, *p* = 0.04), predominantly women (36% vs. 7%, *p* = 0.003), offspring of non-Dutch parents (35% vs. 13%, *p* = 0.06) and those who received EEN orally (27% vs. 10%, *p* = 0.08). Svolos et al. [132] developed a survey which they sent to 41 families of patients who had received treatment with EEN. Despite the difficulties most frequently reported by patients and their families (bad taste of the formula, social isolation, monotony, and excessive duration of treatment), almost 60% would complete another course of EEN in successive flares. Both children and parents agreed that adherence to EEN was lower than to a solid food-based diet (SFD). The majority of respondents would agree to participate in a clinical trial assessing an SFD’s effectiveness for the management of active CD.

## 9. Disadvantages or Points for Improvement of EEN

EEN has shown high efficacy in the short term inducing clinical remission. However, there are no consensual strategies after completing EEN, in Spain [63,131], once the induction period with EEN is completed for 6–8 weeks, 94.2% of the units make a progressive transition to the normal diet in the following 2–4 weeks, 7.6% following their own protocols and the remaining 92.4% without following any protocol. In addition, 5.8% go on a normal diet quickly, without taking into account any order when entering the food groups. There is no data to support either practice. Faiman et al. found no differences between a group of 20 patients who, after EEN, progressively reintroduced the diet (2 new foods every 2 days for 3 weeks while progressively decreasing the amount of polymeric formula) compared to another of 19 patients where after 3 days of low residue diet began an unrestricted diet. In this series, at 6 months, one-third of the patients from each group had developed relapse and a year after EEN, 50% of the patients in group A and 47% of the patients in group B developed relapse [135].

EEN is difficult to carry out and maintain over time as all foods are restricted. EEN is a monotonous strategy that negatively influences the microbiota, decreases the production of short chain fatty acids and microbial diversity by not facilitating exposure to dietary fiber. Although EEN is an effective treatment for inducing remission in paediatric Crohn’s disease, we do not have a sufficiently successful strategy for subsequent maintenance that exempts a significant percentage of patients from requiring biological treatment in the medium term [136].

## 10. Efficacy of Partial Enteral Nutrition (PEN)

Until a few years ago, partial enteral nutrition (PEN) had not been considered an effective strategy to induce remission, and was only considered an option for maintenance of remission in selected patients with very mild disease or generally low risk of relapse associated with other maintenance treatment [129]. In this setting, PEN has proven to be more effective than a normal diet, and as effective as some therapeutic modalities in maintaining remission in patients with inactive CD [137,138]. Wilschanski et al. [139] retrospectively compared 28 children with nocturnal supplementation with elemental formula through NGT with 19 children without supplementation, once they had reached clinical remission. At 12 months, 43% of the supplemented patients had relapsed compared to 79% of those not supplemented. In another series of 8 patients who received 70% of the energy requirements through NGT during variable periods of 1 to 4 months, an increase in growth, a decrease in PCDAI and the need for steroid treatment was observed at 12 months of follow-up [140]. The clinical remission rate was directly proportional to the amount of enteral formula ingested during the supplementation period [139]. Schulman et al. published the results of 42 patients in remission after EEN who received 50% of their calories in the form of PEN. The mean duration of remission was 6 months, with no significant differences with the control group that did not receive PEN [141].

Johnson et al. [82] demonstrated greater efficacy of EEN over PEN to induce remission in children with CD. At 6 weeks of treatment 10/24 (42%, EEN) vs. 4/26 (15%, PEN), (*p* = 0.035) had reached clinical remission. The low response rate to EEN in this study (42%) suggests the presence of other factors that would have conditioned the results. Lee et al. [92], in a prospective, multicentre study, carried out in North America, revealed again the superiority of EEN over PEN (88% vs. 64% according to PCDAI; 45% vs. 14% with faecal calprotectin levels ≤ 250 μg/g).

Based on classic studies such as Johnson’s, other PEN protocols to induce remission have been published with similar results. The protocol of Gupta et al. [142], applied to 23 patients (34% of them on concomitant treatment with corticosteroids), consisted of administering 80–90% of the calories required by the patient through NGT during the night during 8–12 weeks, allowing the patient to take 10-20% of the calories freely during the day. The remission rate was 63%, and a further 16% responded without reaching remission. Up to 65% of the patients reported adverse effects derived from the use of nocturnal EEN (morning vomiting, accidental removal of the tube, non-restorative sleep, abdominal distention, etc.).

## 11. CDED: Today and Tomorrow

Among various diets tested to date, the Crohn’s disease exclusion diet (CDED) is currently the most clinically documented for the management of active CD. CDED is not equivalent to PEN, it is a standardized diet based on the exclusion of certain components, that are abundant in the Western diet that may decrease barrier function, generate dysbiosis or impair the mechanisms for bacterial clearance. To ensure the growth and restoration of the patient’s lean mass, this treatment aims to incorporate a high amount of high-quality protein, reduce the fat content of the diet and incorporate foods rich in complex carbohydrates. For this, readily available natural foods such as chicken, eggs, potatoes, rice, fruits, and vegetables are included, which can be combined with a variable amount of polymeric formula to achieve the energy needs and provide an extra supply of protein, calcium, and vitamin D.

Following these two principles of exclusion and inclusion, the diet is designed in such a way that we can find three different categories of food: mandatory, allowed and not allowed foods. In this way, this treatment ensures the intake of a mandatory amount of beneficial fiber and substrates necessary for the production of SCFA.

CDED consists of several phases that change every 6 weeks, becoming progressively easier for the patient. The first phase is the strictest, since in addition to eliminating the foods and dietary components that trigger inflammation, the amount of certain fruits and vegetables is restricted in order to avoid excessive dietary fiber intake. PEN is added to cover 50% of energy need.

The second phase, lasting another 6 weeks, allows for a greater variety of foods. To give greater flexibility to the diet and improve the quality of life, potentially harmful components and foods not allowed until now, such as gluten, oily fish, red meat, and legumes, are introduced in a controlled manner. A higher fiber intake is also allowed, so that in the last weeks of this phase almost all fruits and vegetables can be included. PEN is reduced to provide 25% of energy needs.

After 12 weeks of dietary treatment, the maintenance phase begins, in which, through free meals, an intermittent and controlled exposure to certain foods not allowed during the first phases is now allowed. In addition, the introduction of free meals allows the diet to be balanced with the aim that this last phase does not have a specific duration, but rather that it becomes a long-term sustainable healthy lifestyle. PEN is recommended to be maintained to cover 25% of energy needs. In the event that throughout the maintenance phase a patient in remission presented inflammatory activity, a reinduction could be evaluated by performing the strictest phases of the diet again for an adaptable period of time according to the response obtained.

This dietary therapy allows to overcome the difficulties posed with EEN and represents a further step with respect to PEN in the treatment of CD, since it allows access to food in a standardized and controlled way, and can be used not only as a strategy for induction being a good option for maintenance of remission in the long term [143]. Moriczi et al. [107] found that at 12 months of follow-up (after EEN + thiopurines), 84/222 (37%) of the patients had been started on therapy with anti-TNF (36 on infliximab and 48 on adalimumab) after a median of 5 months (IQR 1.7–8.7) from finishing EEN.

## 12. Efficacy of CDED

Sigall-Boneh et al. [144] treated 47 patients with a mean age of 16 ± 5.6 years for 6 weeks with a regimen that consisted of administering 50% of their daily energy requirements through a polymeric formula and the other 50% through foods included in the “Crohn’s disease exclusion diet” [144]. The response measured by the Harvey-Bradshaw Index and the PCDAI was 70% (33/47) at 6 weeks. During the following 6 weeks, the amount of polymeric formula was progressively reduced and the recommended diet increased. At the end of 12 weeks, 80% of the patients were still in remission. These results were recently confirmed in a 12-week prospective randomized controlled trial [100]. Children with mild-moderate CD were randomly assigned to a group that received CDED plus 50% of the calories in the form of polymeric formula (Modulen^®^, Nestlé) for 6 weeks (stage 1) followed by CDED along with 25% of the calories using polymeric formula (Modulen^®^, Nestlé) from 7 to 12 weeks (stage 2) (*n* = 40, group 1) or to a group that received EEN for 6 weeks followed by a free diet with 25% of calories in the form polymeric formula (Modulen^®^, Nestlé) during weeks 7 to 12 (*n* = 38, group 2). The patients were evaluated at the beginning of the study and at 3, 6, and 12 weeks. At week 6, 30 (75%) of 40 children who received CDED plus PEN were in steroid-free remission versus 20 (59%) of 34 children who received EEN (*p* = 0.14). At week 12, 28 (75.6%) of the 37 children who received CDED plus PEN were in steroid-free remission compared to 14 (45.1%) of the 31 children who received EEN and then free diet and PEN (*p* = 0.01) [100]. CDED plus PEN was associated with an increase in alpha diversity at 12 weeks compared with those who received 6 weeks of EEN followed with normal diet. Comparison of tolerance and compliance between the two therapeutic regimens demonstrated that tolerance of CDED was higher than that of EEN (97.5% vs. 73.7%, *p* = 0.02) as was compliance (82.5% vs. 76.5%, *p* = 0.52). In a follow up study, the authors concluded that a 3-week trial of dietary therapy (with CDED) can identify children with CD who, with compliance, will be in remission at week 6 [145].

A scenario that frequently occurs in IBD is the loss of response to anti-TNF, with a variable prevalence depending on the series studied [143,146,147]. Sigall-Boneh et al. analyzed the efficacy of CDED to induce remission in patients in whom anti-TNF treatment had failed [148]. A total of 21 patients (10 children and 11 adults) were included. At 6 weeks of treatment with CDED, 6/10 of the children and 7/11 of the adults achieved remission. A significant decrease in CRP levels and an increase in albumin levels were also found. Table 5 summarizes the currently available evidence from the CDED [146,147].

## 13. Advantages of CDED

Mealtime is more important than the food we are eating, excluding a person from this time of socializing and bonding because they cannot eat like the rest of the family can be detrimental and psychologically harmful for the whole family. Coming together regularly for a family meal has been associated with dietary and weight benefits for both adults and children. Positive associations have also been reported between the family meal and improvements in wellbeing, reduced risk-taking behaviors, and fewer disordered eating behaviors in adolescents [149]. EEN implies a radical change in family dynamics affecting the mealtime, a special and relevant moment in Mediterranean culture. In addition, patients who receive EEN experience problems in the social sphere such as integration in school activities, social isolation, concern about not eating solid foods, and the mere fact of feeling different from their friends. CDED allows to reduce these differential aspects that cause so much discomfort in the patient.

PEN as maintenance of remission after the period of EEN is also not a well-accepted practice by patients and parents who advocate dietary advice rather than polymeric formula. Although they attribute beneficial effects to PEN as maintenance therapy, they issue negative comments that must be taken into account: “Made me feel sick and too full”, “Looked like a medicine which put me off”, “Easy to forget to take and bad taste and consistency”, “They were ineffective as he became ill again” or “Suppress my child’s appetite and make him too full” [150]. CDED is an effective alternative to PEN as a maintenance therapy.

An added risk for patients with IBD is following restrictive diets without dietary control. These restrictions are based, in many cases, on personal perceptions, without any scientific basis, with the purpose of either alleviating symptoms or modifying the course of the disease. Diet changes undoubtedly have an impact on the family and social life of these patients. In this sense, the CDED provides a scientific, contrasted, and argued value of dietary restrictions in these patients together with professional dietary support [151,152,153].

According to the report on food consumption in Spain carried out by the Ministry of Agriculture, Fisheries and Food of the Government of Spain, the average expenditure on food per person during 2019 was € 2567.17 (€ 1506.88 at home and € 1060.29 away from home), which yields an average daily expenditure of € 7.03/person. In our country, the price of a specific polymeric formula for Crohn’s disease is € 20/1000 kcal, much higher than the average daily expenditure estimated in Spain [154]. CDED alone or in combination with PEN can be considered as a more cost-effective alternative to EEN and with the same safety and efficacy profile.

## 14. Candidate Selection to CDED

Aside from clinical aspects, there are certain factors specific to the patient and their environment that need to be taken into account before considering the option of CDED as a treatment for CD. Some dietary aspects of the patient, such as very high consumption of high-processed food, being a very selective eater and especially rejection to try new foods, can hinder the development of this treatment in which we need to ensure dietary compliance from the first phase to achieve remission. Therefore, to know these details, it is of particular importance to conduct a complete nutritional assessment, including a detailed dietary interview by a specialized dietitian before starting nutritional therapy [129].

In the case of children and adolescents, another relevant factor, if not the most important, is their family and social settings. It is essential to know the patient’s family situation and structure, the importance given to food at home and make sure that the parents, caregivers and all the people involved in the patient’s feeding know the therapy, understand the importance of the diet in CD and are willing to apply it. In addition, their ability to cook and prepare meals and snacks suitable for CDED should be taken into consideration.

In the event that any of these circumstances, which could preclude the application of this treatment, would arise at the time of diagnosis, it could be considered to start the treatment with EEN while giving the family time and education to prepare before starting CDED. If even then the situation was still not suitable, the patient could be advised to complete the nutritional treatment with EEN while giving dietary advice for the subsequent food reintroduction. After all, CDED should be considered as another option in the treatment of CD that although may present great advantages for the patient, it may not always be the best option in all cases.

## 15. Difficulties and Solutions when Applying CDED

Regarding the CDED, one of the aspects that may concern families when carrying out this treatment is whether the cost of the diet is going to increase the price of their shopping basket by only including natural foods in it. Although price is usually one of the barriers that general population find to be on a healthy diet, if we analyze the cost of the foods included in the CDED (Table 6), it is clear that the expenditure on food per day through the different phases of the CDED alone does not differ from the average spent by a Spaniard on food per day [154] and is much cheaper than EEN.

The time available to spend in the kitchen by parents or caregivers can undoubtedly be one of the limiting factors when carrying out the CDED. In order to facilitate this work, there are several tools available that the dietitian can share, such as mandatory foods check lists, recipes booklets of the different phases, lists of specific products without additives suitable for CDED and where to find them, examples of weekly meal plans, shopping lists, and meal preparation days advices. Another tool that can act as support for patients and families during the whole nutritional therapy is the Modulife^®^ website and app, in which the medical team can invite the patients to register and they can find up to 150 sweet and savory recipes for the different phases, as well as different nutrition and healthy lifestyle tips. It is important to take into consideration that some families may prefer some degree of freedom in making dietary choices while others may require more specific guidance. If required, a personalized meal plan can be prepared for patients based on food preferences and dietary needs.

In a country with such a diverse gastronomic culture as Spain, very different customs and dietary habits can be found even in nearby regions. It is possible that each dietitian needs to adapt the CDED recipes already available to the typical customs of their own area. Occasionally, some families may be an inspiring example by creating new recipes or adapting the existing ones. Aside than encouraging patients to cook new recipes is a great way to stimulate them in the kitchen, it can be a great way of creating a database of local recipes suitable for CDED that the dietitian can collect and share later with the rest of the families. In addition, to motivate families not so imaginative, it would be a very interesting initiative to organize meetings or cooking workshops with patients following the different phases of the diet so that they get to know each other and share their experience, doubts, tips, and tricks.

In a globalized world, it is possible that we meet patients from different countries, who profess different religions or have a different culture. One of the most representative elements of a culture is its food, and this may lead us to think that it can be very difficult to standardize a dietary treatment due to the wide dietary diversity that can be found. In our experience, this situation has not posed any complication with patients from abroad since the great versatility of the mandatory and allowed foods and the fact that a large quantity of condiments for cooking can be used, hence it enables the adaptation of every recipe to the taste of the patient regardless of their traditions.

Every now and then, while explaining the basis of CDED, parents may express concern that the diet includes a certain mandatory food that their children reject or may feel overwhelmed by the size of the mandatory food servings recommended, especially in the case of younger patients. It is important to explain that the portions of the mandatory foods can be adjusted according to the energy and protein needs of the patient. These mandatory foods can also be modified on certain occasions, for example with vegetarian patients or patients with a food allergy, always respecting the dietary components excluded from the diet and ensuring adequate protein intake following the dietitian criteria. It is important to provide the patient with a communication channel (telephone or email) so that the dietitian may solve any doubts or difficulties which may arise between visits to ensure the treatment compliance.

## 16. Multidisciplinary Team: Role of the Dietitian

Adherence to the diet is key to ensure compliance with this treatment and the barriers to achieve it should be discussed at each visit. Diet is one of the issues that most often concerns parents and patients with CD and usually, due to lack of time and specialized staff, is relegated to the background during the consultation with the physician in which so many aspects of the disease (medication, symptom management, etc.) need to be treated.

Two of the best resources that we can provide to patients and their families is time and dedication to explain the basis of the treatment, as well as transmitting encouragement and motivation through the different phases of the diet. This treatment needs continued support and monitoring to ensure the dietary compliance and requirements are met. It is important that patients feel that they have someone to turn when doubts or difficulties arise through the process, so having a support system composed by different professionals (physicians and dietitians) and the patient’s environment is crucial to achieve success. Despite the important role that the dietitian plays within this support system and its recognized importance in the treatment of pediatric CD [129], it should be emphasized that Spain continues to be the only country in Europe that does not incorporate this figure within the public health system, which could be a limiting factor when incorporating this dietary treatment in our country [155].

## 17. Conclusions

The treatment of CD requires attention both to achieve mucosal healing and to optimize growth, while also maintaining proper bone health. EEN is recommended as first-line treatment in luminal CD. The therapeutic mechanisms of EEN are being discovered by advances in the study of the gut microbiota. Although the total exclusion of a normal diet during the time of EEN continues to be of high importance, new modalities of dietary treatment suggest a successful future for the nutritional management of CD. In this sense, CDED is a long-term bet, unlike EEN, it acts on the mechanisms that influence the appearance of inflammation (diet and microbiota), increases the time of clinical remission and promotes healthy lifestyle habits. The development of CDED, which partly minimizes the problems of EEN, has enabled a turnaround in the treatment of pediatric CD.

The CDED has been shown to respond to a frequent demand by patients and their relatives regarding acceptability and compliance. The CDED is as effective as EEN in achieving clinical and biochemical remission but superior to EEN in tolerance and compliance. CDED, unlike EEN, constitutes a long-term strategy for maintaining remission and is nutritionally balanced. By including dietary fiber, CDED corrects the bacterial dysbiosis present in these patients, and is therefore a much more realistic and advanced approach than EEN if complied with adequately. Effective induction with CDED can act as a bridge to starting immune suppression and allows for a period of anti-infectious therapy and optimization of vaccination status. CDED is a long-term strategy that may be used as monotherapy, as combination therapy, for de-escalation of drugs, and as a rescue therapy for refractory patients.

## Figures and Tables

**Table 1:** Dietary factors that potentially affect intestinal barrier mechanisms and host immunity. (Updated from reference [25]).

Dietary Component	Reference	Model	Effects
Natural Components of the Diet			
Gluten	Menta (2019) [29]	Female C57BL/6 mice	In mice with dextran sulfate sodium (DSS)-induced colitis: involvement of the desmosomes, adherent zonule and direct damage to the colon mucosa.
Alcohol	Elamin (2013) [30]	In vitro	Direct damage to the epithelium, increasing intestinal permeability.
Dietary salt	Miranda (2018) [31]	C57BL/6 mice	Decrease in *Lactobacillus* sp. Decrease in butyrate production Increased expression of pro-inflammatory genes such as *Rac1*, *Map2k1*, *Map2k6*, *Atf2*. Suppression of the expression of genes such as *Ccl3*, *Ccl4*, *Cxcl2*, *Cxcr4*, *Ccr7*
Vitamin A	Amit-Romach (2009) [32]	Wistar Rats	Increase of mucus, defensin-6 and TLR
Vitamin D	Vargas-Robles (2019) [33]	WT mice	In mice with colitis induced by dextran sulfate sodium (DSS): maintains the expression of TJ proteins (expression of ZO-1, occludin and claudin-1) and improved barrier function, decreased FITC-dextran permeability and levels circulating LPS.
Zinc	Guthrie (2015) [34]	ZIP14 KO mice	Decreased expression of phosphorylated occludin and claudin-1, and increased claudin-2, maintaining intestinal barrier function.
Flavanones	Liu (2020) [35]	C57BL/6J mice	Increase in ZO-1 and proteins associated with occludin and reduction in serum endotoxin. FXR stimulation with reduced hepatic synthesis of bile acids.
	Stevens (2019) [36]	In vitro	Increase in TEER and decrease in the flow of FITC-dextran, improving intestinal barrier function.
Corn oil	Abulizi (2019) [37]	C57BL/6 mice	Decreases the kinase linked to the integrin, which is essential for barrier function, and decreased expression of various TJ proteins of the intestinal barrier.
High glucose diet	Zhang (2017) [38]	C57BL/6 mice	Increases Th17 differentiation and activation of cytokines.
Fructose	Zhang (2017) [39]	C57BL/6 mice	Mitochondrial dysfunction, increased inflammatory cytokines and intestinal barrier dysfunction.
Aryl hydrocarbon receptor, derived from the digestion of vegetables from the Brasicaceae family	Gao (2018) [40]	Animal models	Necessary for activation and production of Il-22 through innate lymphoid cells type 3 (*ILC3*) and gamma delta intraepithelial T cells in the intestinal barrier
	Smith (2013) [41] Gálvez (2014) [42]	SPF, ASF and GF mice	Reduction of SCFA with reduction of colonic regulatory T cells, especially Th17, important in the pathogenesis of IBD.
Food Additives			
Anthocyanins	Cremonini (2019) [43]	Male C57BL/6J mice Caco-2 cells	Decreases endotoxin levels, increases GLP-2 levels and MUC2 expression.

CD, Crohn’s disease; IFN, interferon; IL, interleukin; Th, T helper; WT, wild-type; ZO, zonula occludens; DSS (dextran sulphate sodium); TJ: tight junctions; LPS: Lipopolysaccharides. This table update the published by Levine [25].

**Table 2:** Dietary factors that potentially affect to microbiome (Updated from reference [25]).

Dietary Component	Reference	Model	Effects
Natural Components of the Diet			
High fat + high sugar diet	Zhang (2012) [44]	C57BL/6J mice	Alteration of the microbiota with a decrease in diversity and an increase in opportunistic pathogens.
Fat	Le Chatelier (2013) [45] Hildebrandt (2009) [46]	Human	Increase in Proteobacteria and Firmicutes and decrease in Bacteroidetes
High fiber diet	Silveira (2017) [47]	BALB/c female mice	High fiber diet protected from acute colitis
Low fiber diet	Hand (2016) [48]	Human	Decrease in intestinal diversity with a predominance of Gram-negatives (Bacteroides, Proteobacteria, Verrucomicrobia) and increase in lipopolysaccharides levels.

This table update the published by Levine [25].

**Table 3:** Clinical remission rates in children with Crohn’s Disease (CD) treated with Exclusive Enteral Nutrition (EEN).

Reference (Year of Publication) (Ref)	N	Formula	T	Remission Criteria	R ^a^
Morin (1980) [15]	4	E	6	CDAI < 150	100%
Sanderson (1987) [68]	8	E	6	Improvement of LSI	88%
Seidman (1991) [69]	10	E	3	CDAI < 150	80%
Seidman (1993) [70]	24	SE	4	CDAI < 150	86%
Thomas (1993) [71]	12	E	4	Improvement of LSI	100%
Beattie (1994) [72]	7	P	8	Improvement of LSI	100%
Ruuska (1994) [73]	10	P	8	PCDAI ≤ 10	90%
Akobeng (2000) [74]	16	P	4	PCDAI < 10	50%
Fell (2000) [75]	29	P	8	PCDAI ≤ 10	79%
Phylactos (2001) [76]	14	P	8	PCDAI ≤ 10	93%
Terrin (2002) [77]	10	SE	8	PCDAI < 10	90%
Ludvigsson (2004) [78]	17	P	6	PCDAI < 10 or decrease 45% or 15 points from baseline	82%
	16	E	6		69%
Afzal (2005) [79]	26	P	8	PCDAI < 20	88%
Knight (2005) [80]	40	E	6	CDAI	90%
	4	P			
Day (2006) [81]	27	P	6–8	PCDAI ≤15	70%
Borrelli (2006) [18]	19	P	10	PCDAI ≤10	79%
Johnson (2006) [82]	24	E	6	PCDAI < 10	41%
Berni Canani (2006) [83]	12	E	8	PCDAI < 10	87%
	13	SE			
	12	P			
Rodrigues (2007) [84]	53	E	6	Not specified ^b^	64%
	45	P			44%
Buchanan (2009) [85]	110	P/E	8	Clinical and biochemical response	80%
Whitten (2010) [86]	23	P	8	PCDAI < 15	69%
Rubio (2011) [65]	106	P	8	PCDAI < 10	81%
Grogan (2012) [87]	20	E	6	PCDAI ≤ 10	70%
	21	P			71%
Lambert (2012) [88]	31	P	6–8	PCDAI < 15	84%
de Bie (2013) [89]	77	P	6	Clinical response	53%
Soo (2013) [90]	36	P/SE	6	PCDAI ≤10	89%
Cameron (2013) [17]	109	P/E	8	PCDAI ≤10	60%
Frivolt (2014) [16]	40	P/E	6–8	wPCDAI < 12.5	95%
Levine (2014) [91]	43	P	6–8	PCDAI < 10	72%
Grover (2014) [19]	28	P	6	PCDAI < 10	79%
Hojsak (2014) [23]	57	P	6–8	PCDAI < 10	84%
Lee (2015) [92]	22	P/E	8	PCDAI ≤ 10	59%
Luo (2015) [93]	13	P	8	PCDAI < 10	69%
Navas (2015) [94]	50	P	6–8	wPCDAI < 12.5	84%
Kim (2016) [95]	66	E	6	PCDAI < 10	88%
Connors (2017) [20]	76	P	8–16	PCDAI < 7.5	87%
Lafferty (2017) [96]	28	P/E	6–8	PCDAI ≤ 10	85%
Luo (2017) [97]	13	P	8	PCDAI ≤ 10	83%
Cohen–Dolev (2018) [98]	60	P	6–8	PCDAI < 10	63%
Pigneur (2019) [99]	13	P	8	Harvey-Bradshaw	100%
Levine (2019) [100]	34	P	6	PCDAI ≤ 10	59%
Logan (2019) [101]	66	P	8	wPCDAI < 12.5	62%
Kang (2019) [102]	19	P	8	PCDAI < 10	65%
Rolandsdotter (2019) [103]	13	P	6	PCDAI ≤ 10	77%
Chan (2020) [95]	13	P	8	PCDAI < 10	69%
Scarpato (2020) [104]	47	P	6–8	PCDAI ≤ 10	68%
Hart (2020) [105]	16	SE	8	PCDAI ≤ 10	93%
Hojsak (2020) [106]	92	P	6–8	PCDAI ≤ 10	77%
Moriczi (2020) [107]	222	P	6–8	wPCDAI < 12.5	83%
Total	2016			IC (95%)	75.7% (73.8–77.5)

CDAI: Crohn’s disease activity index; E: elemental; LSI: Lloyd-Still index; N: number of patients included in the study; P: polymeric; PCDAI, pediatric Crohn’s disease activity index; A: patients reaching remission; SE: semi-elemental; T: time in weeks; wPCDAI: Pediatric Crohn’s disease activity weighted index. ^a^ Intention-to-treat analysis. ^b^ Information obtained from the author.

**Table 4:** Clinical remission rates in adults with CD treated with EEN.

Reference (Year of Publication) (Ref)	N	Formula	T	Remission Criteria	R
O’Morain ^1^ (1984) [14]	11	E	4	H-B	80%
Giaffer ^2^ (1990) [108]	14	P	4	CDAI	36%
	16	E			75%
Malchow ^1^ (1990) [109]	51	P	3–6	CDAI	41%
Lochs ^1^ (1991) [110]	52	P	4–6	CDAI	55%
Rigaud ^2^ (1991) [111]	15	P	4	CDAI	73%
	15	E			66%
Park ^2^ (1991) [112]	7	P	4	Simple activity index	73%
	7	E			69%
Raouf ^2^ (1991) [113]	11	P	3	Simple activity index	71%
	13	E			29%
Lindor ^1^ (1992) [114]	9	E	4	CDAI	33%
González-Huix ^1^ (1993) [115]	15	P	4	Van Hess index	80%
Gorard ^1^ (1993) [116]	22	E	4	Simple activity index	32%
Royall ^2^ (1994) [117]	19	E	3	CDAI	84%
	21	O			75%
Mansfield ^2^ (1995) [118]	22	O	4	CDAI	36%
	22	E			36%
Verma ^2^ (2000) [119]	11	P	4	CDAI	55%
	10	E			80%
Sakurai (2002) [120]	36	P	6	CDAI	70%
Gassull ^1^ (2002) [121]	43	P	4	CDAI	36%
Guo (2013) [122]	13	P	4	CDAI	85%
Hu (2014) [67]	59	O	12	CDAI	81%
Zhao (2015) [123]	40	P/O	4	CDAI	52%
Yang (2017) [66]	41	NE	12	CDAI	80%
Xue (2018) [124]	67	NE	8	CDAI	68%
Xu (2019) [125]	104	NE		H-B	52%
Sharma (2020) [126]	31	P/S-E	2–6	CDAI	80%
Total	797			IC (95%)	60.1% (56.6–63.4)

CDAI: Crohn’s disease activity index. ^1^: Clinical trials comparing with drugs. ^2^: Clinical trials comparing two enteral formulas. E: elemental formula. H-B: Harvey-Bradshaw. NE: not specified. O: oligomeric formula. P: polymeric formula.

**Table 5:** Evidence of Crohn’s Disease Exclusion Diet (CDED) treating patients with Crohn’s disease.

Reference	Study	Patients (*n*)	Remission at Week 6
Sigall-Boneh R (2014) [144]	Retrospective	47	24/33 children 9/14 adults
Sigall-Boneh R (2017) [148]	Retrospective	21	6/10 children 7/11 adults
Levine A (2019) [100]	RCT	40 CDED	30/40 (75%)
Levine A (2020) [143]	Cases series	4	3/3 children/adolescents

RCT: randomized control trial.

**Table 6:** Expenditure on food during the phases of CDED (2000 kcal/day).

	CDED Phase 1	CDED Phase 2	CDED Maintenance Phase
Breakfast	Modulen ^®^(250 mL) 3 Banana pancakes (1 banana + 1 egg)	Modulen^®^(250 mL) Wholewheat bread (1 slice) with olive oil and tomato slices	Modulen^®^(250 mL) Wholewheat bread (1 slice) with olive oil and tomato slices
Snack	Modulen^®^ (350 mL)	Modulen^®^ (250 mL) Carrot oat muffins (1 egg)	Modulen^®^ (250 mL) 1 pear
Lunch	Homemade potato chips Chicken meatballs (100 g) with homemade tomato sauce 1 banana	Chickpeas (20 g) salad with tuna (1 can), 1 boiled egg, avocado (1/3) and sweet potato (1/2) 1 banana	Quinoa salad (20 g) with tomato and onion Grilled salmon (120 g) 1 apple
Snack	Smoothie: Modulen^®^ (350 mL) and apple	Sliced apple with almond butter (10 g)	Yogurt (125 g)
Dinner	Baked chicken breast (150 g) Baked potato and carrot	Homemade beef burger (100 g) Homemade chips potato (1 potato) 1 banana	Spanish omelette (1 egg, 1 potato, onion) Roasted peppers 1 banana
Price	€3.06	€3.93	€3.95
Modulen IBD^®^	€20	€10	€10
